# Discovery and anti-cancer evaluation of two novel non-ATP-competitive FGFR1 inhibitors in non-small-cell lung cancer

**DOI:** 10.1186/s12885-015-1307-9

**Published:** 2015-04-12

**Authors:** Jianzhang Wu, Tao Wei, Qinqin Tang, Bixia Weng, Wulan Li, Xin Jiang, Ting Ding, Xiaokun Li, Guang Liang, Yuepiao Cai, Jiansong Ji

**Affiliations:** 1 Chemical Biology Research Center, College of Pharmaceutical Sciences, Wenzhou Medical University, Wenzhou, Zhejiang 325035 China; 2 College of Information Science and Computer Engineering, Wenzhou Medical Universtiy, Wenzhou, Zhejiang 325035 China; 3 Department of Pharmacy, the Sixth Affiliated Hospital of Wenzhou Medical University, Lishui, Zhejiang 323000 China; 4 Department of Interventional Radiology, The Fifth Affiliated Hospital of Wenzhou Medical University, Lishui, Zhejiang 323000 China

**Keywords:** Fibroblast growth factor receptor 1, Non-small-cell lung cancer, Non-ATP competitive FGFR1 inhibitors, NDGA, Anti-cancer

## Abstract

**Background:**

Fibroblast growth factor receptor 1 (FGFR1) is correlated closely with the occurrence and development of lung cancer. FGFR1 kinase inhibitors have exhibited significant therapeutic effects against non-small-cell lung cancer. Recently, non-ATP competitive FGFR1 inhibitors have attracted extensive attention due to their low side effects.

**Methods:**

Caliper Mobility Shift Assay was used for FGFR1 inhibition test and kinase inhibitory mode study. Hoechst staining and Annexin V/PI staining were used to evaluate the cell apoptosis induction. Western blot were then performed to confirm the intracellular FGFR1 inhibition and apoptotic protein expression. Finally, the anti-tumor effect and mechanism of Af23 and Ad23 was evaluated *in vivo*.

**Results:**

In this study, we designed, synthesized and discovered two novel non-ATP competitive FGFR1 inhibitors, Af23 and Ad23, using NDGA as a leading compound. They had IC_50_ values of 0.6 μM and 1.4 μM against FGFR1 kinase, respectively. The kinase inhibitory assay carried at different ATP concentrations showed that the FGFR1 inhibition mode of both Ad23 and Af23 was non-ATP-competitive. Further, Af23 and Ad23 significantly suppressed FGFR1 phosphorylation and cell proliferation in non-small-cell lung cancer (NSLCLC) H460 cells and induced cell apoptosis. Af23 and Ad23 also showed significant anti-tumor activity in the H460 xenograft mouse model, accompanied with the inhibition of FGFR1, ERK, and AKT phosphorylation without exhibiting toxicity.

**Conclusions:**

These results indicate that Ad23 and Af23 are potential agents for the treatment of non-small-cell lung cancer. This work also provides a structural lead for the design of new non-ATP-competitive FGFR1 inhibitors.

## Background

Lung cancer is the most common cause of death from cancer worldwide, and non-small-cell lung cancer (NSCLC) accounts for 85% of the total incidence [1]. Recently, molecular targeted chemotherapy with RTK inhibitors has been used in clinics for the treatment of NSCLC because of the importance of a series of receptor tyrosine kinases (RTKs) in the development of NSCLC [1,2]. Small-molecule inhibitors of epidermal growth factor receptor (EGFR), such as gefitinib and erlotinib, have been approved by the U.S. Food and Drug Administration (FDA) for the treatment of NSCLC [3,4]. Unfortunately, NSCLC treatment remains challenging because of some problems, including adverse effects and drug resistance associated with the ATP-competitive kinase inhibition mode of the majority of RTK inhibitors [5-7]. The ATP-binding pocket is highly conserved among members of the kinase family, and it is difficult to find selective agents [7,8]. Moreover, the ATP-competitive inhibitors must compete with high intracellular ATP levels leading to a discrepancy between IC_50_s measured by biochemical versus cellular assays. The non-ATP-competitive inhibitors, called type II or type III inhibitors, offer the possibility of overcoming these problems [7,9]. Thus, the development of RTK inhibitors that do not compete with ATP is an urgent need for the treatment of NSCLC.

Fibroblast growth factor receptors (FGFRs) belong to the family of RTK superfamily, and FGFRs, especially FGFR1, was reported to be highly related to the development and progress of NSCLC [1,10-17]. Amplification and activating mutations of FGFR1 have been observed in NSCLC [13,14,17], while silencing the expression of FGFR1 by siRNA or pharmacological inhibition of FGFR1 by small molecules suppresses the development of NSCLC [11,12,16,17]. These observations make FGFRs increasingly a attractive target for the therapeutic intervention in cancer. Two FGFR inhibitors, namely AZD4547 and BGJ398, have entered phase II clinical trials, while more small-molecule inhibitors, such as SU5402, PD173074, TKI-258, and SU6668, have failed in clinical trials due to various complications associated with the side effects caused by the ATP-competitive inhibition mode [8]. To date, only five non-ATP-competitive FGFR inhibitors have been identified, including nordihydroguaiaretic acid (NDGA) [18], NF449 [19], ARQ069 [20], and recently reported A114 and A117 [21]. Despite advances in the field, identifying highly-selective, small-molecule inhibitors that target an inactive conformation or a new domain of FGFR continues to be a significant challenge.

**Figure 1:**
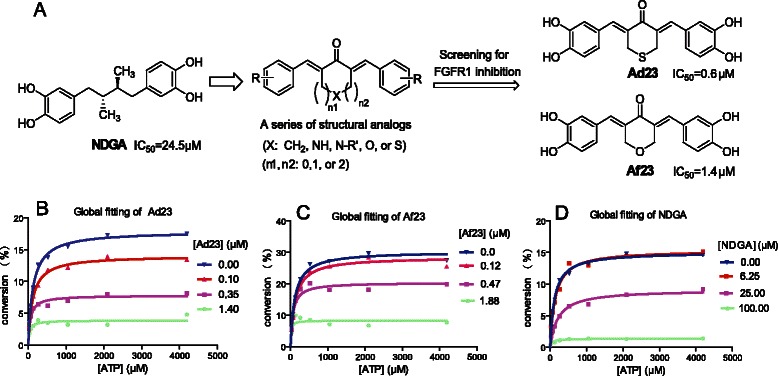
NDGA analogs Af23 and Ad23 inhibited FGFR1 activities in a non-ATP competitive manner. (**A**) The profile of design and FGFR1 kinase inhibition assay of NDGA analogs. FGFR1 kinase inhibition rates of the compounds were evaluated by caliper mobility shift assay, and IC_50_ values were calculated using conversion rates. The data were shown as a mean of 3–5 independent tests. (**B**) Af23 and Ad23 inhibit FGFR1 through a mechanism that is independent of the concentration of ATP. Selective ATP-competitive kinase assay of compounds Ad23 (**B**), Af23 (**C**), and NDGA (**D**) with FGFR1 was carried out through caliper mobility shift assay. The conversion data were fitted with Graphpad for global fitting, using “mixed model inhibition”.

In this report, we characterize two nordihydroguaiaretic acid (NDGA) analogs, i.e., Ad23 and Af23, as two kinase inhibitors that effectively target FGFR1 (Figure 1A). Furthermore, we provide data that indicates these kinase inhibitors have a distinct ATP-independent mode of action. Furthermore, these two compounds have shown excellent anti-cancer activity against NSCLC H460 cells both *in vitro* and *in vivo*. These data further confirm that bisaryl-1,4-dien-3-one structures could be used as non-ATP-competitive FGFR1 inhibitors for the treatment of NSCLC.

## Methods

### Cell lines, compounds, and reagents

NSCLC cell line, H460, was purchased from ATCC (*Manassas, VA*). FGFR1-overexpressing HEK 293 cell lines were kindly gifted by the Institute of Materia Medical, Xi’an Jiaotong University. Various compounds were designed and synthesized in our laboratory, including Af23 (3,5-bis(3,4-dihydroxybenzylidene)-tetrahydropyran-4-one) and Ad23 (3,5-bis(3,4-dihydroxybenzylidene)-tetrahydrothiopyran-4-one) (Figure 1A). Their purity was detected by HPLC (>98.0%) before they were used in biological experiments. The compounds were dissolved in DMSO solution for *in vitro* assay. Their water-soluble formulations for *in vivo* studies were prepared using the pharmaceutical method described previously [22]. All the antibodies were purchased from Santa Cruz Biotechnology (*Santa Cruz, CA*). Hoechst staining kit was purchased from Beyotime Biotech (*Nantong, China*). Recombinant FGFR1 proteins were obtained from Carna Biosciences, Inc. (*Kobe, Japan*).

### Cell-free FGFR1 kinase assays

Using the method described previously in Ref. [21], the FGFR1 kinase inhibition assay of NDGA and its analogs were performed by Caliper Mobility Shift Assay with ATP concentration at its Km value (262 μM). Staurosporine was used as a positive control. For the determination of IC_50_, the compounds were tested in duplicate at 10 concentrations, ranging from 5 nM to 100 μM. Then, conversion data were collected on Caliper EZ reader (*Hopkinton, MA*). The IC_50_ values were obtained by GraphPad Prism 5 (*GraphPad, San Diego, CA*).

### ATP competitive inhibition assay

This experiment was performed to test the relationship between the compounds and ATP in which the concentration of the substrate was constant, while the concentrations of ATP were set at 4192, 2096, 1048, 524, 262, 131, 66, and 33 μM. The global competitive inhibition fit for the compounds was performed based on percent conversion = (Vmax*X)/{km*[(1 + I/Ki)^n^] + X}, where X is the ATP concentration, and n is the Hill coefficient. Specific details of this method were presented in a previous report [21].

### MTT assay

The anti-proliferative activities of compounds were detected by MTT assay. H460 cells were seeded in a 96-well plate with RPMI-1640 medium that contained 0.1% FBS for 24 h. Then, the cells were treated with the compounds at the indicated concentrations (0.74, 2.22, 6.67, 20, and 60 μM) for 72 h. The proliferation of the H460 cells was detected through MTT assay, and the IC_50_ values were calculated by GraphPad software.

### Western blot analysis

Cells or homogenated tumor tissues were lysated. Protein concentrations in all the samples were determined by using the Bradford protein assay kit (*Bio-Rad, Hercules, CA*). The lysates were separated by SDS-PAGE electrophoresis, and electro-transferred to a 0.22-μm polyvinyldene difluoride membrane. After blocking with TBS that contained 5% non-fat milk for 1.5 h at room temperature, the membranes were incubated with different primary antibodies overnight at 4°C. Following the TBST wash, immuno-reactive bands were detected by incubating with respective secondary antibody conjugated with horseradish peroxidase for 1 h. Immuno-reactive bands were visualized by using an ECL kit (*Bio-Rad, Hercules, CA*).

### Hoechest staining

After the H460 cells were incubated with the compounds for 72 h, cells were stained with Hoechst 33342 dye according to the protocol provided with the kit (*Beyotime Biotech, Nantong, China*). The cells were imaged under fluorescent microscope (*Nikon, Tokyo, Japan*), and the pictures were taken at 200× objective.

### Analysis of cell apoptosis

H460 cells were placed in 60-mm plates for 12 h and then treated with NDGA, Ad23, or Af23 at the indicated concentrations for 24 h. Then, the cells were harvested and stained with annexin V and propidium iodide (PI) in the presence of 100 mg/mL of RNAse and 0.1% Triton X-100 for 30 min at 37°C. Flow-cytometric analysis was performed using FACS calibur (*BD Sciences, CA*).

### *In vivo* anti-tumor study

All animal experiments complied with the Wenzhou Medical College Policy on the Care and Use of Laboratory Animals (Wenzhou Medical College Animal Policy and Welfare Committee, Document ID: 201100103). Five to six-week-old athymic nu/nu female BALB/cA mice (18–22 g) were purchased from the Animal Center of the China Pharmaceutical University (Nanjing, China). The animals were housed at a constant room temperature with a 12:12-hr (light: dark) cycle and fed a standard rodent diet and water. H460 cells were harvested and mixed with Matrigel at proportions of 1:1. Then, the cells were injected subcutaneously into the right flank (2 × 10^6^ cells in 200 μL of PBS) of 7-week-old, BALB/cA nude mice. Two days after the H460 cells were injected, the mice were injected intraperitoneally (i.p.) with a water-soluble preparation of either compound Ad23 or compound Af23 in PBS at a dosage of 5 mg/kg/day for 28 days, whereas the control mice were injected with the liposome vehicle in PBS (n = 10 in each group). The volume of the tumors were determined by measuring their length (l) and width (w) and calculated using the formula; V = 0.52 × l × w^2^. The weight of the tumors were recorded on the day the mice were killed.

### Immunohistochemistry analysis

On day 30 after tumor induction, the mice were killed in a CO_2_ chamber, and the tumor tissues were dissected and weighed. Some of the tissues were lysed for protein isolation and then processed for the determination of signaling pathway proteins using Western blot method. A part of harvested tumor tissues were fixed in 10% formalin at room temperature overnight, processed, and embedded in paraffin. The paraffin-embedded tissues were sectioned (5-μm thick) followed by staining with primary antibodies. The signal was detected by biotinylated secondary antibody and developed with 3,3-diaminobenzidine (DAB).

### Statistical analysis

All *in vitro* experiments were repeated at least three times. Data were presented as means ± SD or mean ± SEM. The statistical significance of differences between groups was obtained by the *student’s t test* or ANOVA multiple comparisons in GraphPad Prism 5 (License Number: GPW5-415777-RAG-2191, *GraphPad Software Inc., San Diego, CA*). *P* values less than 0.05 (*p* < 0.05) were considered to be significant.

## Results

### Af23 and Ad23 inhibits FGFR1 kinase via a non-ATP dependent manner

The leading compound NDGA is a natural product isolated from creosote bush. It exhibits multiple pharmacological effects, such as anti-oxidation, anti-inflammation, and anti-tumor [18,23]. Recently, Meyer et al. found that NDGA could inhibit the autophosphorylation of FGFR3 kinase both *in vitro* and *in vivo* [18]. In our previous cell-free assay, we found that the IC_50_ values of NDGA against FGFR1 and FGFR3 were 24.5 and 72.4 μM, respectively, indicating that NDGA exhibits better inhibitory activity against FGFR1 than FGFR3 (Figure 1A). Therefore, using NDGA as a leading compound, we designed and synthesized a series of structural analogs (Figure 1A). Next, we tested the inhibitory activity of synthetic NDGA analogs against FGFR1 kinase by mobility shift assay.

The inhibitory potency of 72 bisaryl-1,4-dien-3-one compounds against FGFR1 kinase was evaluated by *in vitro* kinase assays. Out of the 72 compounds, Ad23 and Af23 were found to exhibit much stronger inhibition against FGFR1 kinase activity than NDGA and other analogs (IC_50_: Ad23,0.6 μM; Af23,1.4 μM) (Figure 1A). Thus these two were chosen for further studies. Subsequently, the kinase inhibition modes of both Ad23 and Af23 were studied. As shown in Figure 1B, the velocity of FGFR1 substrate phosphorylation without inhibitors increases as the ATP concentration increased, and it was reached to the peak at an ATP concentration of 2000 μM. At concentrations greater than the IC_50_ value (1.4 μM for Ad23; 1.88 μM for Af23; 100 μM for NDGA), the kinase activity was decreased by more than 90%, and further increases in the concentration of ATP, even up to 4190 μM, had no effect on the inhibitory potency of the compounds (Figure 1B). These results showed that the inhibition of FGFR1 kinase activity by Ad23, Af23, and NDGA was not dependent on the concentration of ATP. Thus, we obtained two novel non-ATP-competitive FGFR1 inhibitors, i.e., Ad23 and Af23, from the leading NDGA.

### Ad23 and Af23 inhibits the cellular FGFR1 phosphorylation

**Figure 2:**
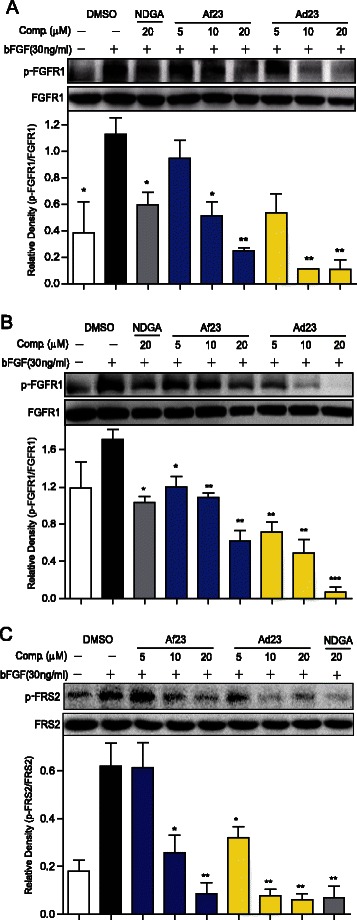
Compounds Ad23 and Af23 inhibited intracellular FGFR1/FRS2 phosphorylation. FGFR1 over-expression 293 cells (**A**) or H460 cells (**B** and **C**) were pretreated with compounds at indicated concentrations or vehicle (0.1% DMSO), respectively. Then, cells were stimulated with bFGF (30 ng/mL) for 10 min, and the phosphorylation levels of FGFR1 (**A** and **B**) and FRS2 (**C**) in cell lysates was measured by western blot analysis. The figures were representative of 3 separate experiments. The column figures show the normalized optical density as a percentage of total protein control. Bars represent the mean ± SEM of 3 independent experiments. Statistical significance relative to bFGF alone group was expressed, *P <0.05, **P < 0.01, ***P < 0.001.

The inhibitory effects of these two compounds on FGFR1 activation were determined in FGFR1-overexpressing 293 cells and human NSCLC H460 cells. As shown in Figures 2A and B, pre-treatment with Ad23 or Af23 dose-dependently reduced the bFGF-induced phosphorylation of FGFR1 in both the cell lines. Also, both Ad23 and Af23 inhibited the phosphorylation of FRS2, a proliferative substrate of FGFR1, in a dose-dependent manner in H460 cells (Figure 2C). Consistent with the cell-free results, Ad23 and Af23 had greater activity than NDGA, and Ad23 showed stronger inhibition than Af23 against cellular FGFR1 phosphorylation.

### Ad23 and Af23 inhibited the proliferation and induced the apoptosis of H460 cell line

**Figure 3:**
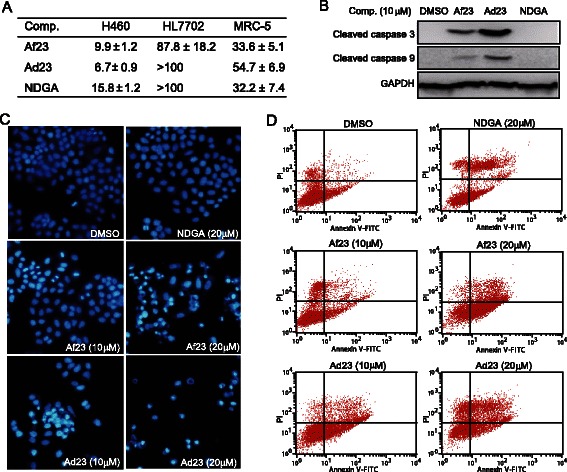
Af23 and Ad23 inhibited proliferation and induced apoptosis in H460 cells. (**A**) H460 cells were treated with Af23, Ad23 or NDGA at different concentration (0.74, 2.22, 6.67, 20, and 60 μM) for 72 h. The viability of H460 cells were detected by MTT assay, and the IC_50_ values of compounds were fitted with GraphPad. (**B**) Effects of Af23 and Ad23 on caspase activation in H460 cells. H460 cells were harvested and lysated after incubated with Af23 (10 μM), Ad23 (10 μM), or NDGA (10 μM) for 12 h. The levels of cleaved caspase-3 and cleaved caspase-9 were determined by western blot analysis. (**C**) Morphological changes and hoechst staining were observed in H460 cells cultured with and without Af23, Ad23, or NDGA at indicated concentrations for 12 h (200×). The figures were representative of more than three separate experiments. **D**. Af23 and Ad23 induced cell apoptosis in H460 cells. H460 cells were treated with Af23, Ad23, or NDGA at indicated concentrations for 24 h, and then stained with Annexin V and PI, followed by detection using flow cytometry. The representative pictures are shown.

Next, the growth inhibition of H460 cells by NDGA, Ad23, and Af23 was studied by MTT assay. Our data showed that Af23, Ad23, and NDGA exhibited marked inhibitory effects against H460 cells (Figure 3A). The inhibitory potencies (IC_50_ values) of Af23 and Ad23 were much greater when compared to NDGA. Further, Western blot analysis showed that the cleavage of caspase-3 and caspase-9 increased after treatment with Ad23 or Af23, indicating that these compounds could induce apoptosis in H460 cells after a 12-h treatment (Figure 3B). Further, Hoechst staining was performed 12 h after treatment with the compounds (Figure 3C). A concentration-dependent increase in the number of cells with nuclear condensation and fragmentation was observed in both the groups. Next, we assessed the effects of Ad23 and Af23 on the induction of apoptosis in H460 cells by flow cytometric analysis. Figure 3D shows that both Ad23 and Af23 dose-dependently increased the H460 apoptosis after 24-h treatment. At a concentrations of 20 μM, both Ad23-treated group (Annexin V^+^/PI^+^, 44.8 ± 7.07%) and Af23-treated group (48.2 ± 11.12%) induced a greater rate of cell apoptosis than NDGA (27.2 ± 5.27%). We have also tested the growth inhibition of Ad23 and Af23 against human liver cells HL7702 and human fetal lung cell line MRC-5 which expresses low level of FGFR1. The results showed that all the IC_50_ values of Ad23, Af23, and NDGA against HL7702 or MRC-5 cells were greater than 30 μM (Figure 3A).

### Ad23 and Af23 significantly suppressed the H460 tumor growth in xenograft mouse model

**Figure 4:**
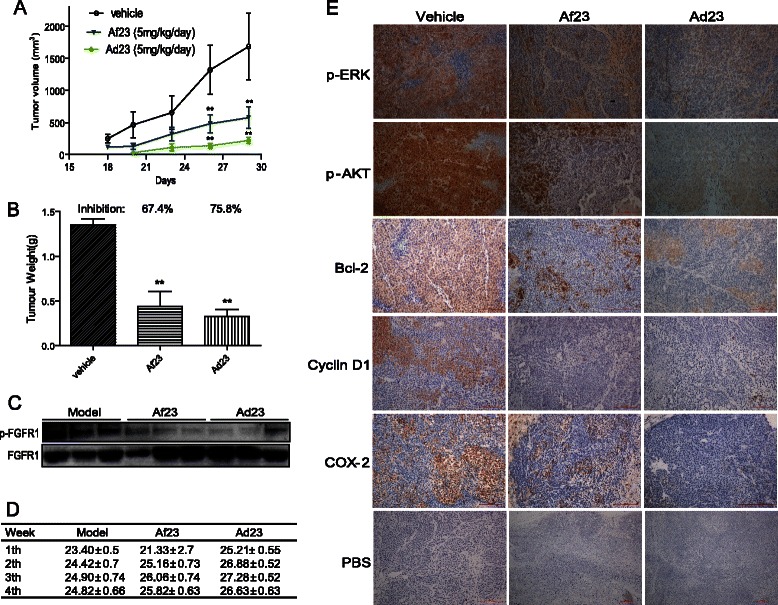
Anti-tumor effects of compounds Af23 and Ad23 in H460 xenograft models. Xenografts were established in nude mice. Two days after the mice were treated with liposome vehicle (once daily, i.p.), Af23 (once daily, i.p., 5 mg/kg/day) or Ad23 (once daily, i.p., 5 mg/kg/day) for 28 days (n = 10 in each group). (**A**) Tumor volume (mm^3^) and (**B**) Tumor weight (g) were recorded (n = 10). Points, mean of 10 mice; bars, SD. **P < 0.01. (**C**) pFGFR1 expressions in tumor tissues were detected by Western Blot with FGFR1 as internal control (3 mice in each group were used); (**D**) Body weight of each group was recorded (n = 10 in each group); (**E**) The levels of p-ERK, p-AKT, Bcl-2, CyclinD1, and COX-2 in tumor tissues were detected by immunohistochemical staining (6 mice in each group were used). Representative pictures are shown.

In order to further assess the anti-tumor activities of the NDGA analogs, we tested the efficacies of Af23 and Ad23 in the H460 xenograft mouse model. Treatment with Af23 or Ad23 for 28 days resulted in significant reduction in tumor volume (Figure 4A). The weight of the tumors were also reduced markedly in the Af23-treated and Ad23-treated groups, with the inhibition rate of 67.4 and 75.8%, respectively (Figure 4B). To evaluate whether the inhibition of tumor growth by Ad23 or Af23 was associated with the inhibition of FGFR1 activity *in vivo*, we analyzed the expression of p-FGFR1 in the tumor tissues. As shown in Figure 4C, Ad23 and Af23 exhibited a significant increased inhibitory effect on FGFR1 phosphorylation in H460 tumor xenografts than the vehicle control tumors. Also, no obvious toxicity was observed in the Af23-treated group or the Ad23-treated group, evidenced by no obvious loss of weight among the two groups during the period of treatment (Figure 4D).

Prior studies have revealed that phosphorylation of FGFR1 can lead to the activation of downstream signaling cascades including ERK and AKT, which plays an important role in the proliferation and survival of cancer cells. Thus, the phosphorylation of ERK and AKT in tumor samples was tested by immunohistochemical assays (Figure 4E). Similarly, these compounds inhibited the FGFR1-downstream ERK and AKT phosphorylation. Finally, the immunochemical data also showed that the expression of Bcl-2, Cyclin D1 and COX-2 was reduced to a far greater extent in the Ad23-treated group and the Af23-treated group than compared to the vehicle-treated group (Figure 4E), indicating that these two FGFR1 inhibitors also induced tumor-cell apoptosis in H460 xenografts.

## Discussion

FGFR1 is highly related to the development of lung cancer [10,12-15,17,24]. Ren et al. identified four NSCLC cell lines and two, newly-established primary lung-cancer cultures that showed high FGFR1 expression levels [24]. They also found that treatment with ponatinib, an FGFR1 inhibitor, could inhibit cell growth in NSCLC cell lines [24]. Terai et al. revealed that the activation of the FGF2-FGFR1 autocrine pathway may be a novel mechanism of acquired resistance to the EGFR inhibitor, gefitinib, in NSCLC [12]. There has been increasing evidence indicating that FGFR1 inhibitors could be a promising candidate for the treatment of NSCLC in clinics. Although a variety of ATP-competitive FGFR1 inhibitors with therapeutic prospects for lung cancer have been identified, most of them failed in pre-clinical or clinical studies because of their low efficacy or high toxicity. At present only two selective FGFR1 inhibitors, i.e., AZD4547 and BGJ398, are being studied in clinical trials (phase II) [8]. The ATP-competitive FGFR1 inhibitors functions by targeting the ATP-binding pocket of FGFR1, which may lead to a decrease in their efficiency when high physiological or intracellular concentrations of ATP exist [8]. In addition, since the ATP-binding site is highly conserved in RTKs, most inhibitors exhibit limited selectivity within RTKs, which induces side effects during treatment, such as nausea, weakness, and elevated blood pressure [25]. For instance, PD173074 and SU5402 failed to enter phase II clinical trials due to their high toxicities [25]. Therefore, the exploration of non-ATP-competitive FGFR1 inhibitors has attracted extensive attention in recent years.

NDGA was reported previously to inhibit FGFR3 kinase [18]. In this study, we found that NDGA exhibited better inhibitory activity against FGFR1 than FGFR3. Using NDGA as a leading compound, we designed and synthesized several NDGA analogs with the basic skeleton of bisaryl-1,4-dien-3-one (Figure 1A). After screening for kinase inhibition, we obtained two FGFR1 inhibitors (Ad23 and Af23) that had inhibitory activities better than compared to NDGA. Interestingly, NDGA and its analogs retained their potency for the inhibition of kinase activity when the concentration of ATP increased, suggesting that the inhibitory effects of these compounds were independent of the concentration of ATP (Figure 1B). So far, ARQ069 is the only published molecule that inhibits FGFR1/2 in a manner that does not compete with ATP. ARQ069 exhibits FGFR1/2 kinase inhibition with IC_50_ values of 0.84 and 1.23 μM, respectively [20]. Although the IC_50_ values of Ad23, Af23, and ARQ069 are at micromolar concentration levels, the observation that the inhibition of FGFR1 autophosphorylation (Figure 2) and the inhibition of the direct FGFR1 downstream substrate (Figure 1A) occur at different dose levels was consistently detectable. A literature search on kinase inhibitors yielded several reports describing that non-ATP-competitive kinase inhibitors may not display identical IC_50_ values with that of classic ATP-competitive inhibitors for the inhibition of both kinase autophosphorylation and downstream signaling pathways [20,24]. Our novel NDGA analogs (Ad23 and Af23) also showed micromole-grade FGFR1 inhibitory effects in a manner that was independent of ATP.

Figure 2 further revealed the anti-FGFR1 ability of Ad23 and Af23 in the cellular levels using FGFR1-overexpressed 293 cells and NSCLC H460 cells. These two compounds also dose-dependently inhibited FRS2 phosphorylation in H460 cells. These data led us to investigate the anti-cancer efficacy of Ad23 and Af23 further. We evaluated the anti-proliferative effects of Ad23 and Af23 *in vitro*. As shown in Figure 3, Ad23 and Af23, as well as NDGA, inhibited cell growth and induced cell apoptosis in NSCLC H460 cells; they also had low toxicities against normal MRC-5 cells and normal human liver cells (HL-7702 cells). Further, we showed the potent anti-tumor ability of these two inhibitors in the H460 xenograft mouse model. Previously, studies on ARQ069, a non-ATP-competitive inhibitor, did not report the *in vivo* anti-tumor activity. In the present study, the data revealed that the compounds Ad23 and Af23 suppressed the phosphorylation of FGFR1 in H460 tumor tissues, thereby inhibited the downstream phosphorylation of ERK and AKT, and reduced the expression of BCL-2, COX-2, and Cyclin D1 (Figure 4). At the same time, we observed that Ad23 and Af23 exhibited high safety *in vivo* (Figure 4D). As already known, the ATP-competitive inhibitory mode leads to the biggest problems (toxicity and side effects) of current FGFR1 inhibitors. Our findings for Ad23 and Af23 indicated that the non-ATP-competitive FGFR1 inhibition might be a new cancer therapeutic alternative with much lower toxicity *in vivo*. However, continued research is needed to examine the underlying FGFR1-binding mechanism and preclinical evaluation of these two compounds. In spite of the predicted “DFG-OUT” docking model, it is unclear how Ad23/Af23 exactly binds to the FGFR1 kinase domain or other domains, which needs to be demonstrated by X-ray diffraction-based structural biology study. Also, it is important to test the RTK-inhibitory selectivity of these two compounds at both molecular and cellular levels in the future.

## Conclusion

Given the critical importance of FGFR1 in the pathogenesis and development of NSCLC, this study identified two novel, non-ATP-competitive inhibitors of FGFR1 kinase, i.e., Ad23 and Af23, both of which exhibited good anti-tumor activity *in vitro* and *in vivo*. These two compounds were shown to have the potential to be developed as novel agents for the treatment of NSCLC. The results of the present study indicate that Ad23 and Af23deserve further studies both in the pre-clinical evaluation and in the field of medicinal chemistry for developing structurally different and more effective bisaryl-1,4-dien-3-one-containing FGFR1 inhibitors.
